# Identification of key ferroptosis genes in diabetic retinopathy based on bioinformatics analysis

**DOI:** 10.1371/journal.pone.0280548

**Published:** 2023-01-23

**Authors:** Yan Huang, Jun Peng, Qiuhua Liang

**Affiliations:** 1 Clinical College of Jining Medical University, Jining, China; 2 The First Hospital of Hebei Medical University, Shijiazhuang, China; 3 Department of Endocrinology, Affiliated Hospital of Jining Medical University, Jining, China; North Carolina State University, UNITED STATES

## Abstract

**Objectives:**

Diabetic retinopathy (DR) is a retinal microvascular disease associated with diabetes. Ferroptosis is a new type of programmed cell death that may participate in the occurrence and development of DR. Therefore, this study aimed to identify the DR ferroptosis-related genes by bioinformatics methods.

**Methods:**

The RNAseq data of DR and healthy control retinas were downloaded from the gene expression synthesis (GEO) database and analyzed using the R package DESeq2. The key modules were obtained using the WGCNA algorithm, and their genes were intersected with ferroptosis-related genes in the FerrDb database to obtain differentially expressed ferroptosis-related genes (DE-FRGs). Enrichment analysis was conducted to understand the function and enrichment pathways of ferroptosis genes in DR, and hub genes were identified by protein-protein interaction (PPI) analysis. The diagnostic accuracy of hub genes for DR was evaluated according to the area under the ROC curve. The TRRUST database was then used to predict the regulatory relationship between transcription factors and target genes, with the mirDIP, ENCORI, RNAnter, RNA22, miRWalk and miRDB databases used to predict the regulatory relationship between miRNAs and target genes. Finally, another data set was used to verify the hub genes.

**Results:**

In total, 52 ferroptosis-related DEGs (43 up-regulated and 9 down-regulated) were identified using 15 DR samples and 3 control samples and were shown to be significantly enriched in the intrinsic apoptotic signaling pathway, autophagosome, iron ion binding and p53 signaling pathway. Seven hub genes of DR ferroptosis were identified through PPI network analysis, but only HMOX1 and PTGS2 were differentially expressed in another data set. The miRNAs prediction showed that hsa-miR-873-5p was the key miRNA regulating HMOX1, while hsa-miR-624-5p and hsa-miR-542-3p were the key miRNAs regulating PTGS2. Furthermore, HMOX1 and PTGS2 were regulated by 13 and 20 transcription factors, respectively.

**Conclusion:**

The hub genes HMOX1 and PTGS2, and their associated transcription factors and miRNAs, may be involved in ferroptosis in diabetic retinopathy. Therefore, the specific mechanism is worthy of further investigation.

## 1. Introduction

Diabetic retinopathy (DR) is a retinal microvascular disease associated with diabetes, accounting for an estimated 4.8% of global blindness, and has become the main cause of acquired visual loss in middle-aged people worldwide [1]. DR is classified as non-proliferative or proliferative DR according to the development stage [2]. Oxidative stress and inflammation play an important role in DR pathophysiology [3] but the pathogenesis and mechanism of DR remain unclear.

Ferroptosis is a new mode of programmed cell death that is different from apoptosis and involves high iron-dependent lipid peroxidation [4]. The cumulation of lipid peroxides leads to the loss of selective cell membrane permeability [5]. However, glutathione peroxidase 4 (GPX4) plays an important role in protecting cell membrane from peroxidation damage that can prevent the cell membrane from being affected by oxidation [6]. When ferroptosis occurs, the antioxidant glutathione is exhausted, leading to GPX4 failure and ultimately, fatal accumulation of lipid peroxides [7].

The high glucose environment can inhibit the growth of human retinal capillary endothelial cells, and ferroptosis can increase this effect, possibly related to GPX4 ubiquitination promoted by TRIM46 [8]. The fatty acid binding protein 4 (FABP4) can inhibit lipid peroxidation and oxidative stress by regulating ferroptosis, thereby reducing retinal damage in DR [9]. In addition, non-coding RNA (ncRNA), such as circular RNA and miRNA, also participate in the ferroptosis of DR [10, 11]; thus, ferroptosis is closely related to DR initiation and progression, but the underlying mechanism of ferroptosis in DR remains unknown.

Therefore, this study identified differentially expressed genes (DEGs) in the retina of DR patients and normal retinas and then matched these DEGs to the ferroptosis dataset to acquire differentially expressed ferroptosis-related genes (DE-FRGs) to determine the mechanism of DR development. Our findings reveal the role of ferroptosis in DR, which may become a target of clinical drug therapy in the future.

## 2. Materials and methods

### 2.1. Data collection and acquisition of ferroptosis-related gene

GEO (https://www.ncbi.nlm.nih.gov/geo/) belongs to public databases. Users can download relevant data for free for research and publish relevant articles [12]. In this study, the GSE102485 mRNA expression profile dataset was downloaded from GEO. GSE102485 contains 19 specimens of diabetic retinopathy and 3 normal retinas and is based on the GPL18573 platform (Illumina NextSeq 500, Homo sapiens). The public FerrDb (http://www.zhounan.org/ferrdb) database for ferroptosis-related genes (FRGs) that promote, inhibit, or mark ferroptosis was also searched, and after the removal of duplicate genes, 232 FRGs were finally obtained for subsequent analysis.

### 2.2. Identification of DEGs

R software (version 4.1.3; https://www.r-project.org/) and the Bioconductor software package (http://www.bioconductor.org/)) were used to correct and analyze the original data. RNA-seq data processing and normalization were performed using DESeq2 packages. Principal component analysis (PCA) was used to verify the repeatability of the GSE102485 data. The standard of statistical significance was |log2FC| > 1 and adjusted p < 0.05. DEGs were visualized in a volcano map based on the "ggplot2" software package.

### 2.3. Weighted gene co-expression network analysis

Based on the scale-free topology criterion, a co-expression network of DEGs was constructed. First, all thresholds were analyzed using the WGCNA software package in R software to determine the soft threshold power. Then, a weighted co-expression network was constructed, and the classifier was clustered into multiple modules with different colored labels to determine the correlation between each module and the control. The module most related to diabetes was considered the key module for further analysis.

### 2.4. Differential expression analysis of ferroptosis-related genes

The differentially expressed ferroptosis-related genes (DE-FRGs) were cross genes between key module genes and FRGs. At the same time, determine whether DE-FRGs are genes of single gene symbols. We used the "Venn Diagram" package of R software to draw Venn diagram to show the number of DE-FRGs. Used the "ggplot2" package to display the expression of DE-FRGs in the heat-map.

### 2.5. GO and KEGG analysis of DE-FGRs

Functional annotations and pathway enrichment for GO biological processes and KEGG annotation were performed using the “Cluster analyzer” software package. The enrichment results were sorted according to the adjusted P value. The enrichment bar chart shown the first 10 results.

### 2.6. Construction of a PPI network of DE-FRGs and hub gene identification

The PPI network was constructed using the search tool of STRING online database (https://cn.string-db.org/) and visualized by Cytoscape software. The top 10 genes of the PPI network were determined as the hub genes, which were calculated based on the maximal clique centrality (MCC), maximum neighborhood component (MNC), degree, edge percolated component (EPC) and Closeness algorithms y utilizing the cytohubba plug-in. The expression of hub genes was visualized in a boxplot, and the area under the curve (AUC) was measured by ROC curve analysis of hub genes. The black line diagram was drawn using the “circlize” package of R software to show the hub genes correlations.

### 2.7. TFs-Genes–miRNAs interaction networks

Transcriptional Regulatory Relationships Unraveled by Sentence-based Text mining (TRRUST) is the largest freely available database of human transcription factors (TFs) and genes interactions [13]. We obtained the TFs regulating hub gene from TRRUST database. Then mirDIP, ENCORI, RNAnter, RNA22, miRWalk and miRDB online databases were used to construct the miRNAs-genes cooperative regulation network. The TFs-genes-miRNAs network was visualized by Cytoscape software.

### 2.8. Verification of hub genes

The GSE94019 database from the GEO database was used to verify the accuracy of the identified hub genes. The "limma" package of R software was used to calibrate the data set, and differences between DR and controls were compared using a Wilcoxon test (* p-value < 0.05 and ** p-value < 0.01). The box graph was drawn using R software.

## 3. Results

### 3.1. Identification of DEGs

**Fig 1:**
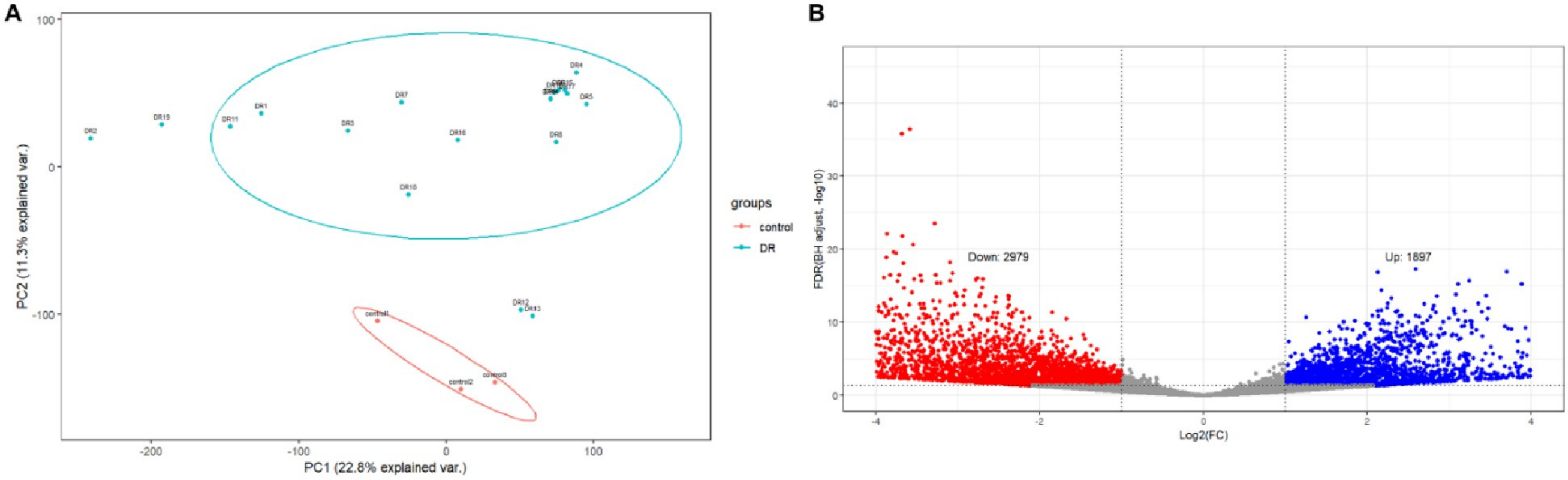
DEGs in DR and control samples. (A) PCA for GSE102485. Red, control; Green, DR. (B) Volcano of the 4876 DEGs. Red, up-regulation; Blue down-regulation.

PCA was used to detect whether biological replicates in the same treatment group were clustered together and whether samples in different treatment groups were separated from each other. The analysis results showed that the GSE102485 data set had well repeatability (Fig 1A). It was worth noting that PCA also showed that four DR samples were outliers, so they were excluded from the analysis. Subsequently, the genes of 15 DR retinal tissues and 3 normal retinal tissues were used for differential gene analysis. Used the adjusted p value < 0.05 and |log2FC| > 1 as criterion. In all, 4876 DEGs were identified, including 2979 up-regulated genes and 1897 down-regulated genes. The differential expression of these 4876 DEGs in the DR and the control groups is shown in the volcanic map (Fig 1B).

### 3.2. WGCNA analysis

**Fig 2:**
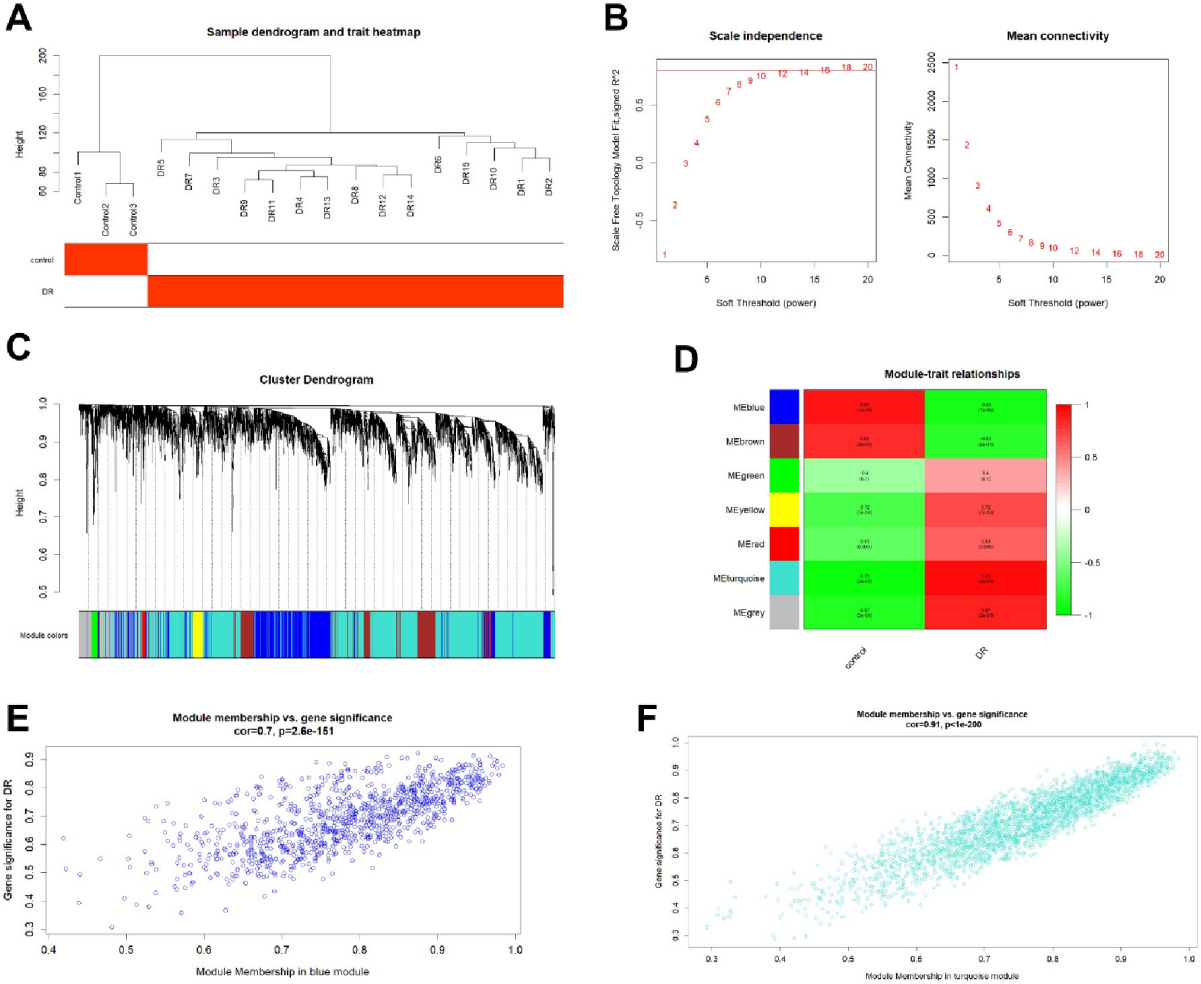
WGCNA of DEGs. (A) Sample clustering tree to check for outliers. (B) Scale-free networks and average connectivity for the soft threshold. (C) Clusters based on the topological overlap matrix. (D) The heat map showed the correlation of module eigengenes with DR patients or controls. (E-F) Scatter plots showing the correlations between MM and GS in the blue and turquoise modules.

The WGCNA package in R software was used to further process 4876 identified DEGs. When building the sample tree, there were no abnormal samples, so no samples were removed (Fig 2A). The soft threshold power of 18 was used to establish a scale-free co-expression network (scale-free R2 > 0.8) (Fig 2B). The clusters were divided into seven modules, blue, brown, green, red, yellow, turquoise and green, with a minimum module size of ≥ 30 (Fig 2C). The correlation between each module and DR was determined (Fig 2D), showing that blue (-0.88, P < 0.0001) and turquoise (0.95, P < 0.0001) were the most negative and positive modules related to DR, respectively. These two modules were the first two modules significantly related to clinical characteristics. The blue (cor = 0.7, P = 2.6e-151) and turquoise (cor = 0.91, P < 1e-200) modules showed a significant positive correlation between MM and GS of the target genes (Fig 2E and 2F). Therefore, blue module and turquoise module were considered as key modules.

### 3.3. Identification of DE-FRGs

**Fig 3:**
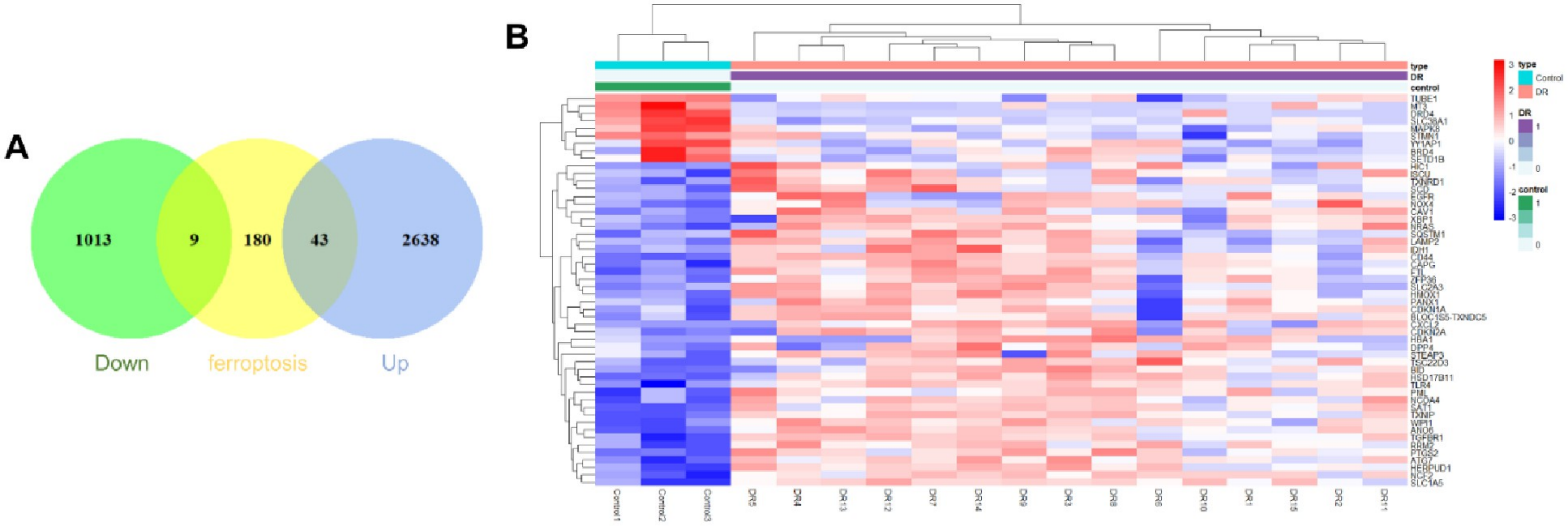
Acquisition of DE-FRGs and their differential expression in the DR and control groups. (A) 3703 DEGs were intersected with the ferroptosis dataset to obtain 52 DE-FRGs. Down (green) represents the number of genes in the negative correlation module, ferroptosis (yellow) represents the number of ferroptosis genes, and Up (cobalt blue) represents the number of genes in the positive correlation module. (B) The heat map showed the distribution of 52 DE-FRGs in the DR and control groups. Blue represents down-regulated genes; red represents up-regulated genes.

The data of 232 genes from the FerrDb database were crossed with the genes of key modules to determine DE-FRGs to identify. A total of 43 positively-related genes and 9 negative related genes were found. These 52 DE-FRGs are all single gene symbols, and the heat-map and Venn diagram are shown in Fig 3. These DE-FRGs were further classified as ferroptosis driver, suppressor, or marker genes (Table 1).

**Table 1:** The DE-FRGs were classified as ferroptosis driver, suppressor, or marker.

Suppressor	Driver	Marker
PML, BRD4, ISCU, CDKN1A, ZFP36, CD44, CAV1, SCD, LAMP2, SQSTM1, HMOX1	WIPI1, MAPK8, YY1AP1, EGFR, BID, SLC38A1, NOX4, ATG7, ANO6, NRAS, CDKN2A, TLR4, TGFBR1, PANX1, SAT1, DPP4, IDH1, SLC1A5, NCOA4, HMOX1	MT3, TUBE1, SETD1B, CXCL2, HSD17B11, CAPG, STMN1, DRD4, HBA1, TXNRD1, XBP1, BLOC1S5-TXNDC5, FTL, RRM2, TSC22D3, SLC2A3, STEAP3, PTGS2, HERPUD1, HMOX1, NCF2, TXNIP, HIC1

### 3.4. GO and KEGG analysis of DE-FRGs

**Fig 4:**
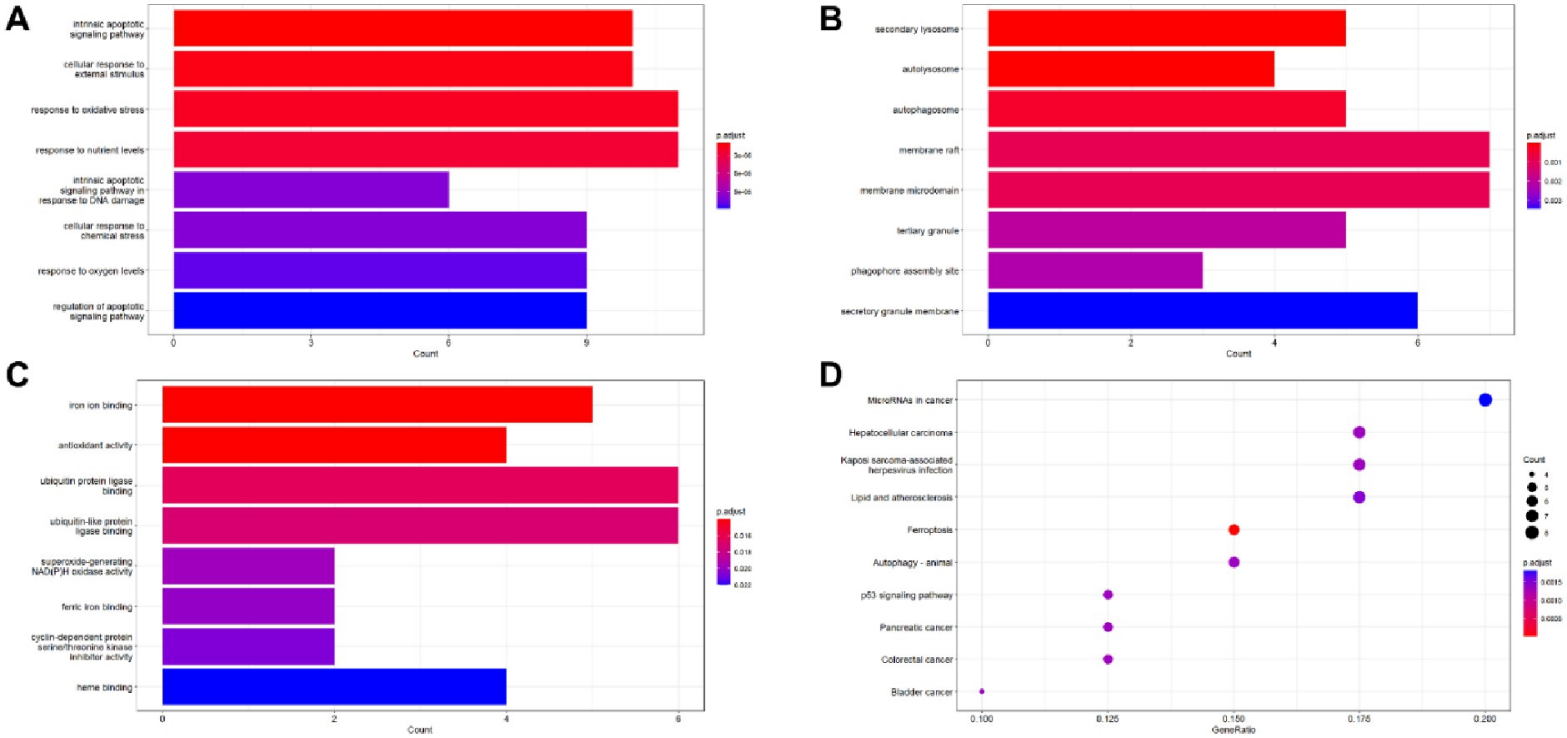
Functional enrichment analysis of DE-FRGs. (A-C) The GO analysis shows BP (Biological process), CC (Cellular component) and MF (Molecular function) respectively. (D) KEGG pathway analysis.

DE-FRGs were enriched and analyzed by R software to define their underlying physiological functions. The enrichment histogram shows the top 10 results. The most significant enrichment terms were the intrinsic apoptotic signaling pathway, cellular response to external stimulus, response to oxidative stress, response to nutrient levels(biological process) (Fig 4A), secondary lysosome, the autolysosome, autophagosome, membrane raft (cellular component) (Fig 4B), iron ion binding, antioxidant activity, ubiquitin protein ligase binding, ubiquitin-like protein ligase binding (molecular function)(Fig 4C). As expected, the KEGG pathway analysis showed that these DE-FRGs were significantly enriched in ferroptosis, the p53 signaling pathway, and endocrine resistance (Fig 4D).

### 3.5. PPI network of DE-FRGs

**Fig 5:**
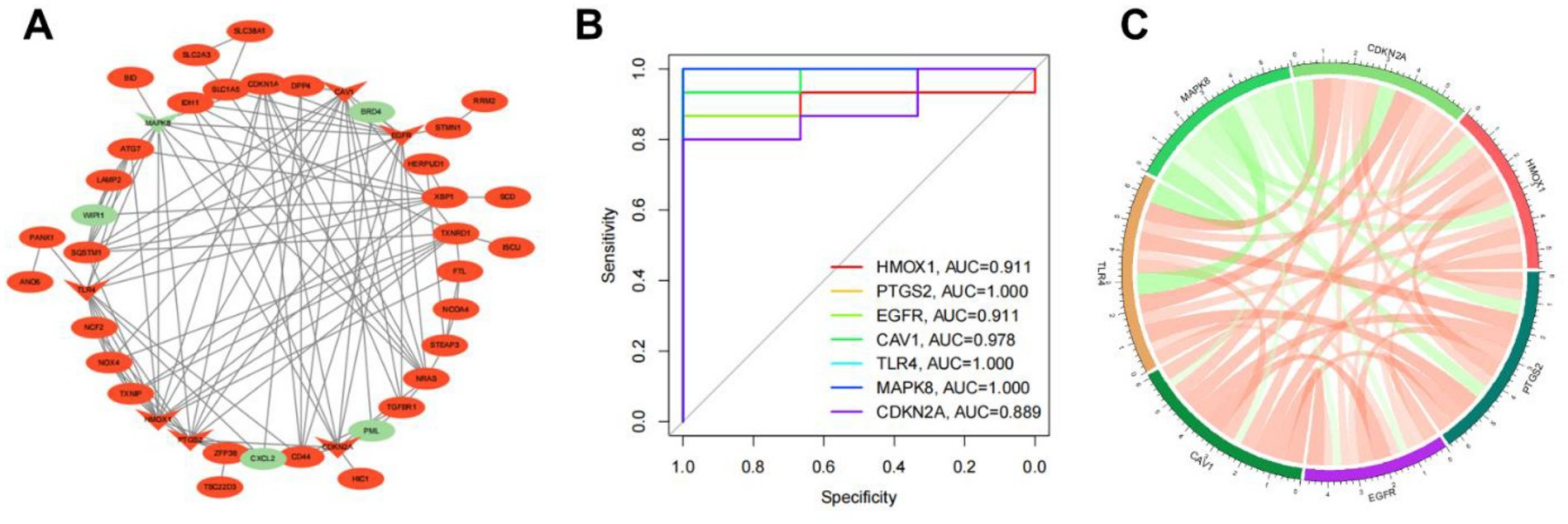
PPI Network of DE-FRGs and hub gene detection. (A) PPI network of 52 DE-FRGs. Green represents downregulated genes, and red represents upregulated genes. V shape represents the hub genes; ellipse represents others. (B) ROC curves of the seven genes for the diagnosis when distinguishing DR from normal. (C)Red represents positive correlation, green represents negative correlation, and the darker the color or the thicker the line, the higher the correlation intensity.

Protein-Protein interaction networks were created to demonstrate the interactions between DE-FRGs (Fig 5A). We get a PPI network with 42 nodes and 115 edges. Ten of the 52 genes are not related to other molecules and do not form a molecular network. There are 47 up-regulated genes and 5 down-regulated genes in PPI network. Table 2 shows the first 10 central genes obtained in by five algorithms (MCC, MNC, Degree, EPC, Closeness). The overlapping genes in the five algorithms are selected as hub genes. They are HMOX1, PTGS2, EGFR, CAV1, TLR4, MAPK8, CDKN2A. HMOX1, PTGS2, EGFR, CAV1, TLR4, and CDKN2A are up-regulated genes. MAPK8 is a down-regulated gene.

**Table 2:** Top ten hub genes obtained by five algorithms of Cytohubba.

MCC	MNC	Degree	EPC	Closeness
PTGS2	HMOX1	EGFR	HMOX1	EGFR
HMOX1	PTGS2	HMOX1	PTGS2	HMOX1
EGFR	EGFR	PTGS2	TLR4	TLR4
TLR4	CAV1	TLR4	EGFR	PTGS2
MAPK8	TLR4	CAV1	CAV1	CAV1
CAV1	MAPK8	MAPK8	MAPK8	MAPK8
CDKN1A	SQSTM1	CDKN2A	CD44	TXNRD1
NOX4	CD44	TXNRD1	SQSTM1	SQSTM1
CDKN2A	CDKN2A	SQSTM1	CDKN1A	CDKN2A
CD44	CDKN1A	XBP1	CDKN2A	NRAS

### 3.6. Hub gene detection

We discuss the diagnostic ability of these seven genes in different patients, and draw the ROC curve. The results show that the AUC of HMOX1, PTGS2, EGFR, CAV1, TLR4, MAPK8 and CDKN2A were 0.911, 1.000, 0.911, 0.978, 1.000, 1.000 and 0.889 respectively when distinguishing DR patients from normal controls (Fig 5B). The results show that 7 genes as new biomarkers have high diagnostic accuracy. The chord diagram shows that there is a strong correlation between the seven genes (Fig 5C).

### 3.7. Further miRNAs and TFs interaction and mining

**Fig 6:**
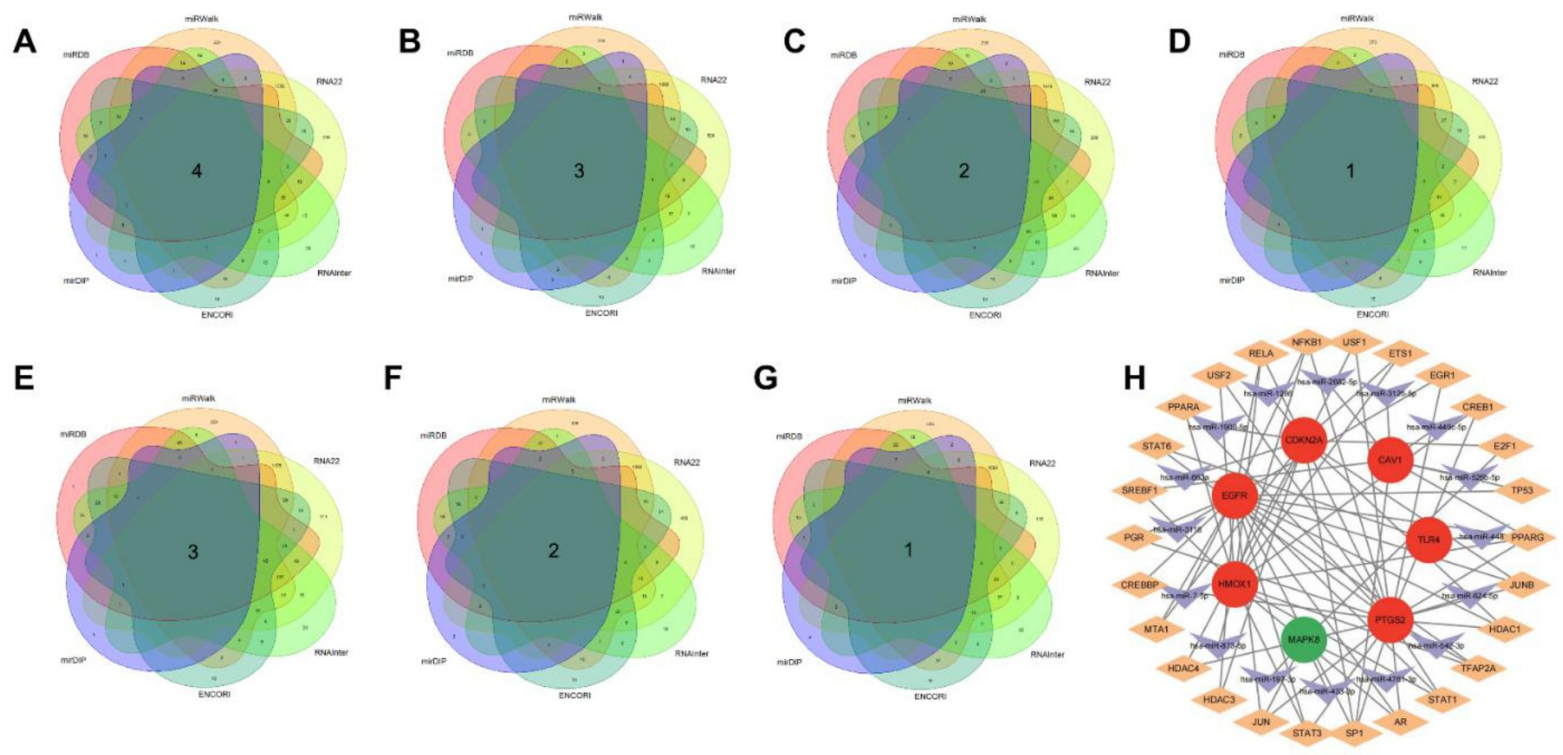
Network of miRNAs and TFs interacting with hub genes. (A-G) The number of miRNAs that CAV1, CDKN2A, EGFR, HMOX1, MAPK8, PTGS2 and TLR4 coexist in the five databases. (H) Network of miRNAs-genes-TFs interacting with hub genes. V shape represents miRNAs; Diamond represents TFs; Ellipse represents hub genes, red represents up-regulated genes, and green represents down-regulated gene.

MiRNAs-genes-TFs interactions were collected using network analysis. Hub genes (HMOX1, PTGS2, EGFR, CAV1, TLR4, MAPK8, CDKN2A) were screened for miRNAs and TFs identification (Fig 6H). CAV1 is regulated by 4 miRNAs, CDKN2A by 3 miRNAs, EGFR by 2 miRNAs, HMOX1 by 1 miRNA, MAPK8 by 3 miRNAs, PTGS2 by 2 miRNAs and TLR4 by 1 miRNA (Table 3). CAV1 is regulated by 4 TFs, CDKN2A by 8 TFs, EGFR by 17 TFs, HMOX1 by 13 TFs, MAPK8 by 2 TFs, PTGS2 by 20 TFs, and TLR4 by 1 TF (Table 4). There was a common hub gene in these TFs regulatory networks, indicating that there is a high degree of interaction between TFs and hub genes.

**Table 3:** Key miRNAs that regulate hub genes predicted by six databases.

hsa-miR-2682-5p hsa-miR-3126-5p hsa-miR-449c-5p hsa-miR-526b-5p	hsa-miR-663a hsa-miR-1908-5p hsa-miR-1286	hsa-miR-7-5p hsa-miR-3118	hsa-miR-873-5p	hsa-miR-4761-3p hsa-miR-433-3p hsa-miR-197-3p	hsa-miR-624-5p hsa-miR-542-3p	hsa-miR-448
**CAV1**	**CDKN2A**	**EGFR**	**HMOX1**	**MAPK8**	**PTGS2**	**TLR4**

**Table 4:** Key TFs that regulate hub genes predicted by TRRUST database.

Key TFs	Description	P value	List of overlapped genes
PPARG	peroxisome proliferator-activated receptor gamma	4.74E-09	HMOX1, PTGS2, EGFR, CAV1
JUNB	jun B proto-oncogene	1.42E-08	PTGS2, CDKN2A, EGFR
HDAC1	histone deacetylase 1	1.77E-06	EGFR, CDKN2A, PTGS2
TFAP2A	transcription factor AP-2 alpha (activating enhancer binding protein 2 alpha)	1.77E-06	PTGS2, EGFR, HMOX1
STAT1	signal transducer and activator of transcription 1, 91kDa	2.94E-06	PTGS2, EGFR, HMOX1
AR	androgen receptor	4.00E-06	HMOX1, EGFR, PTGS2
SP1	Sp1 transcription factor	1.27E-05	EGFR, CDKN2A, PTGS2, CAV1
STAT3	signal transducer and activator of transcription 3 (acute-phase response factor)	1.43E-05	PTGS2, EGFR, HMOX1
JUN	jun proto-oncogene	1.65E-05	MAPK8, EGFR, PTGS2
HDAC3	histone deacetylase 3	3.24E-05	EGFR, CDKN2A
HDAC4	histone deacetylase 4	3.24E-05	CDKN2A, PTGS2
MTA1	metastasis associated 1	3.24E-05	CDKN2A, EGFR
CREBBP	CREB binding protein	3.52E-05	PTGS2, EGFR
PGR	progesterone receptor	3.52E-05	PTGS2, EGFR
SREBF1	sterol regulatory element binding transcription factor 1	4.12E-05	HMOX1, CAV1
STAT6	signal transducer and activator of transcription 6, interleukin-4 induced	7.39E-05	TLR4, PTGS2
PPARA	peroxisome proliferator-activated receptor alpha	8.68E-05	HMOX1, PTGS2
USF2	upstream transcription factor 2, c-fos interacting	0.000127	PTGS2, HMOX1
RELA	v-rel reticuloendotheliosis viral oncogene homolog A (avian)	0.000134	PTGS2, EGFR, HMOX1
NFKB1	nuclear factor of kappa light polypeptide gene enhancer in B-cells 1	0.000137	EGFR, PTGS2, HMOX1
USF1	upstream transcription factor 1	0.000243	PTGS2, HMOX1
ETS1	v-ets erythroblastosis virus E26 oncogene homolog 1 (avian)	0.000359	HMOX1, CDKN2A
EGR1	early growth response 1	0.000445	EGFR, PTGS2
CREB1	cAMP responsive element binding protein 1	0.000465	HMOX1, PTGS2
E2F1	E2F transcription factor 1	0.00103	CDKN2A, MAPK8
TP53	tumor protein p53	0.00153	CAV1, EGFR

### 3.8 Verification of hub genes

**Fig 7:**
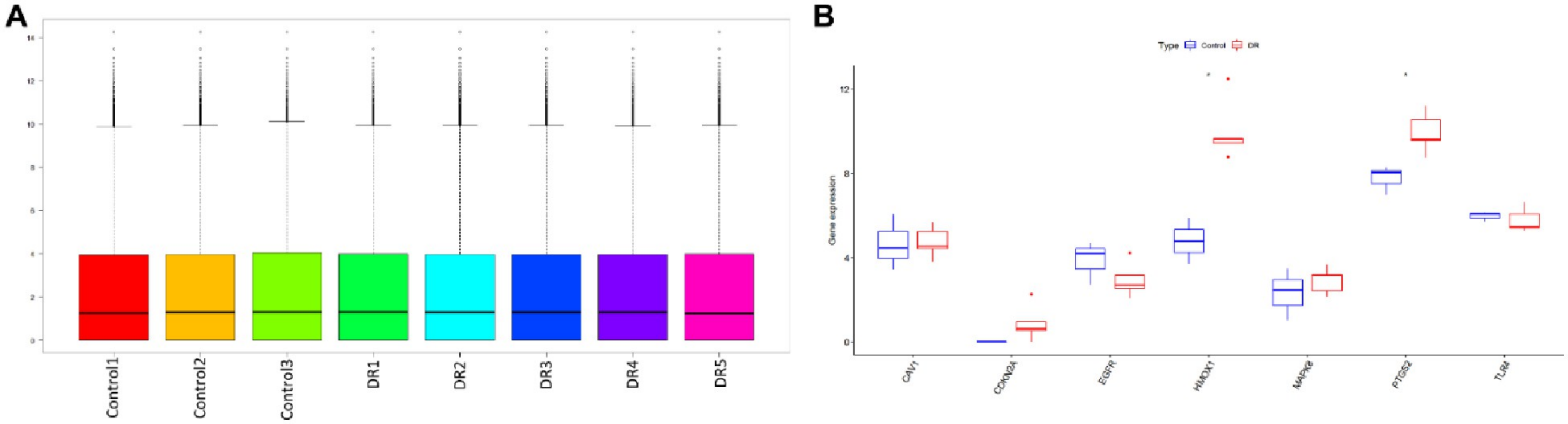
Gene expression of samples. (A) The values of each group centered on the median. (B) Expression of hub genes in each group. *p < 0.05.

The “ggpubr” package of R software was used to view the data distribution, and the values of each group centered on the median are shown in Fig 7A. This shows that the data in GSE94019 are uniform and comparable, so the samples passed the quality test. The comparison of the DEGs in the DR and the control groups showed that only two genes (HMOX1 and PTGS2) were different. The expression of hub genes in each group is shown in Fig 7B.

## 4. Discussion

Diabetic retinopathy (DR) is a special microvascular complication of diabetes and an important cause of blindness in many countries [14]. In the early stage, that is, non-proliferative DR, the blood vessels in the retina are weakened, resulting in fluid leakage, macular swelling, and eventually blurred vision, whereas, in late proliferative DR, abnormal neovascularization on the surface of the retina seriously impairs vision [8]. Ii is predicted that by 2040, the number of DR patients will reach 191million globally, bringing great pressure to individuals, families, and society [15]. Therefore, elucidating the pathogenesis of DR is important to reduce the risk of blindness in diabetic patients.

Ferroptosis is a new mode of programmed cell death involving the accumulation of lipid peroxides that damage the cell membrane and is considered different from apoptosis, necrosis, and autophagy [16]. Li et al. used bioinformatics methods to identify the key genes of ferroptosis-related to Calcic aortic valve disease and proved in vitro that these hub genes are differentially expressed in Calcic aortic valve disease and normal people [17]. Previous studies have shown that ferroptosis is associated with aging retinopathy [18], light-induced retinal degeneration [19], retinitis pigmentosa [20], and glaucoma damage [21]. Therefore, we applied bioinformatics methods to explore hub genes involved in DR ferroptosis.

The present study showed that the expression of ferroptosis genes was different between the diabetic retinopathy and normal control groups. PCA indicated that the samples of DR group and control group were obviously different. There were 52 DE-FRGs that were involved in apoptosis, iron binding, autophagy, and other processes. The PPI network identified seven hub genes related to ferroptosis in DR, including HMOX1, PTGS2, EGFR, CAV1, TLR4, MAPK8, and CDKN2A. However, after verification with another data set, only HMOX1 and PTGS2 were shown to be differentially expressed between the DR and control groups. In addition, when testing the diagnostic accuracy of hub genes, the AUC of PTGS2 was equal to 1, possibly due to the small number of samples included in the study. The AUC of a subset of genes obtained using machine learning in the study by Li et al also equals 1 [22], which may indicate that an AUC of 1 is a relatively reliable diagnostic model in small-sample studies. In the future, further verification should be conducted using a larger sample size.

Heme oxygenase-1 (HMOX1)is the rate-limiting enzyme involved in the heme oxygenase reaction pathway, degrading heme into carbon monoxide (CO), ferrous (Fe^2+^) and biliverdin IXα [23]. The increased HMOX1 in a mouse model reduced the neuronal cell death induced by oxidative stress [24]; however, excessive activation of HMOX1 can cause glioma cell death [25]. In retinopathy, the protective or damaging effect of HMOX1 on the retina depends on the expression of HMOX1 [26]. Our study shows that the high HMOX1 expression may be related to DR, but the role of HMOX1 in DR is still unclear. Prostaglandin endoperoxide synthase (PTGS) is a rate-limiting enzyme in the arachidonic acid synthesis of prostaglandins (PGs). It is expressed exists widely in mouse, rat and human retinas, including prostaglandin endoperoxide synthase-1 and prostaglandin endoperoxide synthase-2 (PTGS1 and PTGS2) [27]. PTGS2, which can be induced by cytokines, mitogens and endotoxins, is the immediate early genetic product of inflammation [28] and may be involved in DR by regulating the anti-angiogenic factor TSP-1 and its receptor CD36 on endothelial cells [29]. Our study suggests that PTGS2 may be involved in the process of ferroptosis causing DR. Based on the close relationship between the high glucose environment and inflammation, PTGS2 and inflammation, the specific mechanism of PTGS2 participating in DR is worthy of further study.

MicroRNA (miRNA) is a small non-coding RNA involved in post-transcriptional gene regulation by degrading or inhibiting the translation of target genes [30]. TFs as modular proteins can bind to the DNA-binding domain in the promoter region of target genes to regulate transcription [31]. In our study, hsa-miR-873-5p was the key miRNA regulating HMOX1, while hsa-miR-624-5p and hsa-miR-542-3p were the key miRNAs regulating PTGS2. TFs prediction showed that HMOX1 and PTGS2 were regulated by 13 and 20 TFs respectively. It has been shown that miR-542-3p promotes the rapid degradation of PTGS2 mRNA, which partly supports our bioinformatics prediction [32]. MiR-624-5p [33–35] and miR-873-5p [36–38] have been studied in cancer, but their role in DR has not been investigated. According to the analysis of published literature, relevant TFs PPARG [39, 40], STAT1 [41], AR [42], SP1 [43], STAT3 [44], RELA [45] and EGR1 [46] are closely related to DR. The above studies reflect that our predictions are relatively reliable. Our research suggests that TFs genes miRNAs connection may play a role in DR, but a lot of research is still needed to explore its specific mechanism.

## 5. Conclusion

In conclusion, we found that HMOX1 and PTGS2 are hub genes involved in ferroptosis process of diabetic retinopathy. Our study is helpful to increase the understanding of researchers on the pathogenesis of diabetic retinopathy. However, how these ferroptosis-related genes play a role needs to be explored by future in vivo and in vitro experiments.
